# Inhibition of melanoma using a nanoceria-based prolonged oxygen-generating phototherapy hydrogel

**DOI:** 10.3389/fonc.2023.1126094

**Published:** 2023-03-16

**Authors:** Lidong Zhang, Xiaoguang Liu, Yinghua Mao, Shu Rong, Yonghong Chen, Yong Qi, Zhipeng Cai, Hong Li

**Affiliations:** ^1^ Institute of Military Preventive Medicine, Army Medical University (Third Military Medical University), Chongqing, China; ^2^ Department of Gynecology, Women’s Hospital of Nanjing Medical University (Nanjing Maternity and Child Health Care Hospital), Nanjing, China; ^3^ Centre for Diseases Prevention and Control of Eastern Theater, Nanjing, China; ^4^ Huadong Research Institute for Medicine and Biotechniques, Nanjing, China

**Keywords:** photodynamic therapy, hydrogel, melanoma, nanaoceria, hypoxia alleviation

## Abstract

Tumor hypoxic environment is an inevitable obstacle for photodynamic therapy (PDT) of melanoma. Herein, a multifunctional oxygen-generating hydrogel loaded with hyaluronic acid-chlorin e6 modified nanoceria and calcium peroxide (Gel-HCeC-CaO_2_) was developed for the phototherapy of melanoma. The thermo-sensitive hydrogel could act as a sustained drug delivery system to accumulate photosensitizers (chlorin e6, Ce6) around the tumor, followed by cellular uptake mediated by nanocarrier and hyaluronic acid (HA) targeting. The moderate sustained oxygen generation in the hydrogel was produced by the reaction of calcium peroxide (CaO_2_) with infiltrated H_2_O in the presence of catalase mimetic nanoceria. The developed Gel-HCeC-CaO_2_ could efficiently alleviate the hypoxia microenvironment of tumors as indicated by the expression of hypoxia-inducible factor -1α (HIF-1α), meeting the “once injection, repeat irradiation” strategy and enhanced PDT efficacy. The prolonged oxygen-generating phototherapy hydrogel system provided a new strategy for tumor hypoxia alleviation and PDT.

## 1 Introduction

The global morbidity and mortality of melanoma are increased dramatically, which is still one of the severe threats to public health (1–3). However, traditional tumor treatment often confronts obstacles due to visible toxic side effects and drug resistance (4). Recently, synergetic photodynamic therapy (PDT) has been introduced to overcome melanoma and has shown great potential in the field of tumor therapy (5, 6). As a noninvasive technology, PDT addresses spectacular advantages such as high localized tissue damage and minimal side effects (7). In the PDT process, photosensitizers such as chlorin e6 (Ce6) accumulated in tumor tissues are usually activated and energized by a specified light, resulting in reactive oxygen species (ROS) generation and singlet oxygen (^1^O_2_) in the presence of biological substrates and oxygen (8). Therefore, oxygen and photosensitizer contents in tumor tissues are restrictive factors for PDT reaction and cytotoxicity (9). However, the therapeutic efficacy of PDT is often unsatisfactory because of the hypoxic microenvironment in most malignant tumors and rapid drug metabolism-induced short-term effects (10–12). It is of great significance to alleviate hypoxia and prolong photosensitizer supply in the tumor environment for enhancing PDT efficacy.

Several efforts have been conducted to overcome tumor hypoxia, such as water splitting (13, 14), respiratory inhibitor (15, 16), O_2_-evolving agents (17, 18), catalase (19, 20), and nanoscale metal-organic framework (21, 22). Among them, catalase could effectively improve the efficacy of oxygen supply and PDT treatment because of the catalytic decomposition of elevated H_2_O_2_ in the tumor environment (23, 24). However, the poor stability, cost, and limited catalyst capacity restrict the application of natural catalase. These limitations have emerged the nanoenzymes, which are defined as nanomaterials with enzyme-like characteristics (25, 26). Numbers of nanomaterials based on iron oxides (27), copper oxides (28, 29), vanadium oxides (30, 31), and noble metals (Au, Ag, Pt, Pd) (32–36) have been reported to mimic catalase activity.

Cerium nanoparticles (CeNPs) are a remarkably versatile rare earth nanomaterial with excellent catalytic activities (37, 38). It has been reported to mimic multiple types of enzymes due to the electron shuttle between their mixed oxidation states (Ce^3+^/Ce^4+^) and has emerged as a fascinating material in biological fields (39). Recently, we demonstrated that chemically cytotoxic and oxygen-carrying CeNPs could act as a nanocarrier to deliver photosensitizers into tumor cells, resulting in enhanced PDT efficacy (40, 41). In this study, to obtain sufficient oxygen generation for PDT, the CeNPs nanocarrier was introduced into the system and used as an ideal renewable catalase-like enzyme to catalyze the reaction of CaO_2_ and H_2_O. Herein, we designed a prolonged oxygen-generating phototherapy hydrogel system (Gel-HCeC-CaO_2_) to improve the therapeutic efficacy of PDT in treating melanoma.

## 2 Materials and methods

### 2.1 Chemicals and reagents

Poloxamer P407 (F127) and Poloxamer P188 (F68) were purchased from BASF,Germany. H_2_O_2_ (technical grade, 30%) was offered by Aladdin,China. CaO_2_, Cerium (III) nitrate hexahydrate, Docusate sodium(AOT), and Alendronate sodium were offered by Sigma Aldrich,USA. Chlorin e6 (Ce6) was offered by Frontier Scientific, Inc,USA. HA with a molecular weight (MW) of 17kDa was purchased from Lifecore Co. Dulbecco’s modified Eagle’s medium (DMEM), Fetal bovine serum (FBS) and Trypsin-EDTA (0.25%) were purchased from Gibco,USA. The Cell counting kit-8 (CCK-8 kit) were purchased from Dojindo,Japan. Trizol reagent was offered by Invitrogen,USA. PrimeScript RT Master Mix was purchased from Takara,Japan. SYBR Green PCR Master Mix was purchased from Applied Biosystems,USA. All chemicals and reagents were of analytical grade.

### 2.2 Synthesis of materials

The naked cerium oxide nanoparticles (MCeNPs), cerium oxide nanoparticles-alendronate (CeNPs-AL), hyaluronic acid-chlorin e6 modified nanoceria (HCeC), and Gel-HCeC-CaO_2_ were prepared as described in the literature (17, 42).

MCeNPs: 0.4548 g AOT and 1.5 ml Ce(NO_3_)_3_·6H_2_O aqueous solution(0.1 M) were added to 30 ml methylbenzene under potent stirring for 45 min (4500 rpm/min). Then H_2_O_2_ was added using a pipette tip slowly under potent stirring for 1 h (4500 rpm/min).

CeNPs-AL: 20 mg AL,200 mg Na_2_CO_3,_ and 10 ml MCeNPs were added to 5 ml ddH_2_O under potent stirring for 24 h, then centrifugal (5 min,3000 rpm). After that, we used a dialysis bag (Thermo, 10kDa) to dialyze the acquired CNPs-AL for 24 h.

HCeC: 0.5 mL Ce(NO_3_)_3_·6H_2_O aqueous solution(0.1 M) were added to 10 mL HA aqueous solution(5 mg·ml^-1^) at 37 °C under potent stirring. After 15 min, 0.8 ml sodium hydroxide (NaOH) (1M) was added to the mixture and stirred for 30 min. Then, 400 μl Ce6 (10 mM) was quickly added and stirred for 5.5 h. After that, the dialysis bag (Thermo, 10 kDa) was used to dialyze the acquired HCeC for 24 h in dark and rinsed with water repeatedly in a sleeve tube (Millipore, 30 kD). The acquired HCeC were dispersed in ddH_2_O (HCe not contained Ce6).

Gel-HCeC-CaO_2_: 0.26 g Poloxamer407 (F127),7.5 mg CaO_2_ were mixed in a 1 mL bottle, then 400 μL PoloxamerP188 (F68) and 313 μL HCeC were added to it. At last, ddH_2_O was added to 1 mL and acquired Gel-HCeC-CaO_2_ system (Gel not contained CaO_2_ and HCeC, Gel-CaO_2_ only included CaO_2_).

### 2.3 Characterization of HCe and HCeC

The zeta potential of the HCe and HCeC were determined using dynamic light scattering(Invitrogen, America). The hydration of nanoparticles was determined by dynamic light scattering (DLS) analysis. The HCe and HCeC were characterized by HR-TEM (FEI, America) at an accelerating voltage of 200 kV. The fluorescence spectra of Ce6, HCe, and HCe6 were determined by Fluoromax-4 spectrofluorometer (Horiba Jobin Yvon Inc, France) at 405nm excitation light. The absorption spectra of Ce6, HCe, and HCeC solution were determined by a UV spectrophotometer (KAIAO, China). Moreover, the HCe and HCeC were cultured with ddH_2_OˎPBS ˎDMEMˎDMEM+ 10% FBS for 24 h to observe the stability. The photosensitivity of HCeC was detected by singlet oxygen sensor green reagent (SOSG). And we mixed the HCeC with 50 mM H_2_O_2_ to show the CAT-like activity of the HCeC.

### 2.4 Sol-Gel transition behavior of the Gel-HCeC-CaO_2_ system

1 mL Gel and Gel-HCeC-CaO_2_ solution were added into a 10 mL bottle, then put the bottle into a 37 °C water bath at a different time until the solution transformed to gel, recording the time (43). The test tube inverting method was used to determine the sol-gel transition behavior of the hydrogel (44), 1 mL Gel and Gel-HCeC-CaO_2_ solution was placed in a 10 mL bottle at -20 °C, 4 °C, 25 °C, and 37 °C to observe the solution transformed to gel. 1 mL Gel-HCeC-CaO_2_ solution was placed in a 10 mL bottle and incubated at 37 °C for 5 min to gel. 1 mL PBS (pH = 7.4, 37 °C) and CaCl_2_ solution were gently added into the bottle as a release medium. The bottles were shaken at the speed of 50 rpm in a thermostatic shaker (HENGZI, China) at 37°C while the weight of the gel was measured every 20 min.

### 2.5 Oxygen generation of the Gel-HCeC-CaO_2_ system

N_2_ was injected into 30 mL PBS for 30 min to produce the deoxygenated PBS. The deoxygenated PBS was divided into two groups, and the PH was adjusted by concentrated hydrochloric acid to 5.4 and 7.2, respectively. 5 mL Gel-HCeC-CaO_2_ solution were put into a 50 mL centrifuge tube and incubated at 37 °C for 5 min to gel. Then, 30 mL deoxygenated PBS (PH=7.2/5.4, 37 °C) was added into Gel-HCeC-CaO_2_ solution. Afterward, we added 10 mL cooking oil isolate deoxygenated PBS and air. We used the Dissolved Oxygen Meters (Mettler, Switzerland) to detect oxygen generation.

### 2.6 Cell experiment and design

We obtained the murine melanoma cells (B16F10) lines from the Chinese Academy of Sciences cell bank. Cells were cultured in RPMI 1640 culture medium containing 10% FBS and 1% penicillin-streptomycin in a controlled environment (37 °C, 95% air, 5% CO_2_).

Cell migration assay: B16F10 cells were seeded into 6-well plates and allowed to grow 80%-90% confluence. We used a sterile 20 μL pipette tip to scrape the confluent monolayer to form a cell-free zone. Then, B16F10 cells were incubated with HCeC (1 μg ·mL^−1^) or Gel-HCeC-CaO_2_ (1 μg ·mL^−1^) for 24 h and irradiated by the 660 nm laser(200 mW/cm^2^) irradiation for 5 min (45). Cells were photographed at 0, 24, and 48 h with a light microscope (Olympus, Japan).

Cytotoxicity assays: B16F10 cells were seeded at an initial density of 1 × 10^4^ in 200 µl RPMI 1640 medium. After 24 h, cells were incubated with HCeC (0,0.125,1.25,12.5,25 μg·mL^-1^, dark, laser) or Gel-HCeC-CaO_2_ (0,0.005,0.05,0.5,1 μg·mL^−1^, dark, Laser). The laser group received the 660 nm laser (200 mW/cm^2^) irradiation for 5 min after incubation at 24 h. After 48 h, we added 10 μL CCK-8 solution to each well and detected the absorbance at 450 nm.

### 2.7 Animal experiment and design

Animal model: All BALB/c-nu mice animal procedures were approved by SPF Biotechnology Co., Ltd in our experiments. The melanoma cancer model was generated by subcutaneous injection of B16F10 cell (5×10^5^) suspended in PBS into the flank region of the right back of BALB/c mice and allowed them to grow into solid tumors.

Biodistribution of Gel-HCeC-CaO_2_ in B16F10 tumor-bearing mice: Mice were injected with 100μL Ce6, HCeC, and Gel-HCeC-CaO_2_ after the tumor volume reached about~65 mm^3^. We used an *in vivo* imaging system (Perkin Elmer, America) to observe the mice at 0,4, and 20 h after injection. And the major organs such as the liver, spleen, kidney, heart, lung, and tumor were excised for further imaging analysis.

Antitumor on orthotopic B16F10 model: When the tumor size achieved ~65 mm^3^, B16F10 tumor-bearing mice were randomly divided into eight groups (n=6/group): PBS (100μL,dark),Gel-CaO_2_ (100μL,dark),HCeC (100μL,dark),HCeC (100μL,laser),HCeC (100μL,laser*2),Gel-HCeC-CaO_2_ (100μL,dark),Gel-HCeC-CaO_2_ (100μL,laser), and Gel-HCeC-CaO_2_ (100μL,laser*2). The laser group was irradiated with 660 nm laser (200 mW/cm^2^) irradiation for 5 min at 4 h or 20 h. And we recorded the body weights and tumor volumes of mice every 2 days (46). The tumor volumes were measured with a digital caliper and calculated as the following formula: width^2^×length×0.5.

Histological analysis and anti-metastatic activity of mice: The tumors of all mice were collected and subjected to the H&E staining or TUNEL assays after the mice were sacrificed. We used a light microscope (Olympus, Japan) to observe the histological change. At the same time, the lungs of mice were also excised and photographed (47).

### 2.8 Quantitative polymerase chain reaction

The oligonucleotide primers were designed by Sangon Biotech (Shanghai, China) and listed in **Table 1**. The total RNA was extracted by TRIzol reagent and quantified by NanoDrop2000 spectrophotometer (Thermo Fisher Scientific, USA). The PrimeScript RT Master Mix and SYBR Green PCR Master Mix were used for performing the qPCR (48).

**Table 1:** Primer sequences of target genes for qPCR.

Gene	Species	Forward primer sequence (5'-3')	Reverse primer sequence (5'-3')
β-actin	mouse	CCACCATGTACCCAGGCATT	CGGACTCATCGTACTCCTGC
TNF-α	mouse	GAATGAAGTGCACCCTAACAAG	GAGGAATGGGTTCACAAATCAG

### 2.9 Statistical analysis

The data were expressed as mean values ± SEM, including at least three biological replicates. The Student’s t-test and one-way analysis of variance (ANOVA) were utilized to determine the statistical significance of differences among groups. Statistical values are indicated according to the following scale: ⁎p < 0.05, ⁎⁎p < 0.01, ⁎⁎⁎p < 0.001. All statistical analyses were performed by SPSS 19.0 software.

## 3 Results

### 3.1 Characterization of HCe and HCeC

**Figure 1:**
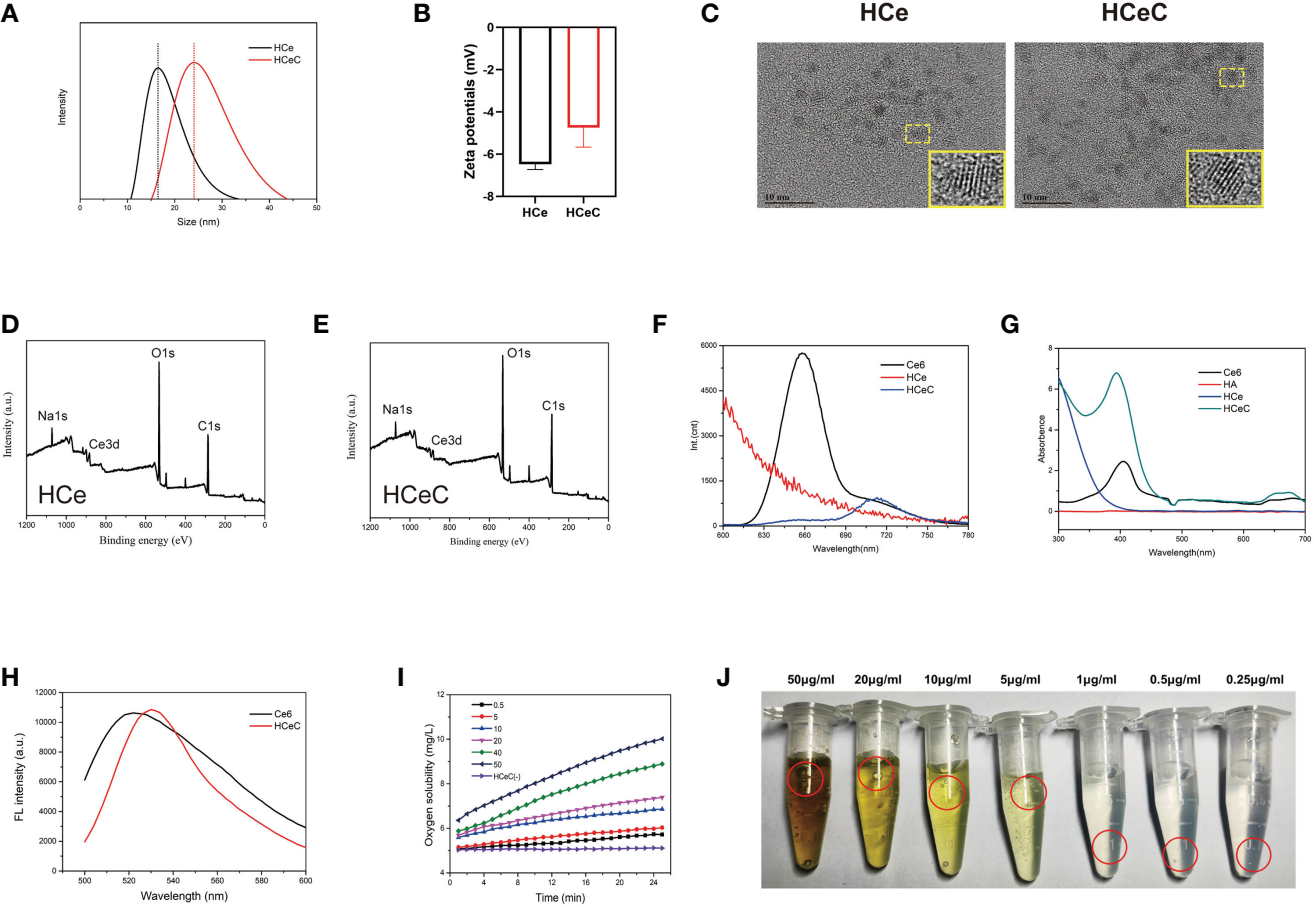
Characterization of HCe and HCeC. **(A)** DLS of HCe and HCeC. **(B)** Zeta potential of HCe and HCeC. **(C)** TEM of HCe and HCeC. **(D)** XPS analysis of HCe. **(E)** XPS analysis of HCeC. **(F)** Fluorescence spectra of Ce6, HCe, and HCeC. **(G)** UV–vis absorption spectroscopy of Ce6, HA, HCe and HCeC. **(H)** Photosensitivity of HCeC. **(I, J)** The CAT-like activity of HCeC.

The MCeNPs was synthesized by the microemulsion method (49). As shown in **Supplementary Figure 1**, the solution was milky white in the process of magnetic stirrers, which stratified overnight. The upper popcorn liquid was a toluene organic phase containing MCeNPs and the underlying dark yellow liquid was an aqueous phase containing CeNPS-Al. Due to the lack of effective surface protection, MCNPs was agglomerated in solution and cleared by the endothelial reticular system (ERS) in the body. Therefore, HCe and HCeC were synthesized by probing the interaction between CeNPs and HA to improve the stability of MCeNPs. To measure the size distribution of HCe and HCeC, we performed the DLS. As shown in **Figure 1A**, the average diameter of HCeC was approximately 25.60 nm while HCe was about 19.86 nm, because the Ce6 molecule increased the molecular diameter of HCe. And the zeta potential of the HCeC was approximately -6mV and the HCe was -5.6mV in **Figure 1B**, owing to the presence of the carboxyl group in Ce6. To examine the structure of the HCe and HCeC, the detailed morphology was characterized by HR-TEM. Due to the protective effect of HA (50), we found the size of HCe and HCeC are about 2-3nm and 3-4nm, showing good dispersion in water in **Figure 1C**. The Ce^3+^/Ce^4+^ ratio is crucial for the enzyme-mimetic activity, the X-ray photoelectron spectroscopy (XPS) analysis of HCe and HCeC showed high Ce^3+^/Ce^4+^ ratio in **Figures 1D, E** and **Supplementary Figures 2A–C**. Besides, the results of Fourier transform infrared spectra (FT-IR) showed that HA was conjugated to nanoceria successfully in **Supplementary Figure 2D**.

To verify that Ce6 was linked to HCe, the fluorescence spectra of Ce6, HCe, and HCeC were determined by a fluorescence spectrometer at 405 nm excitation light in **Figure 1F**, and the characteristic fluorescence peaks of Ce6 were between 640-690nm. Owing to the electron transfer between HCe and Ce6, HCeC showed significant fluorescence quenching compared with Ce6. The successful preparation of HCeC was validated by UV–vis absorption spectroscopy in **Figure 1G**. The characteristic absorption peak of Ce6 was at 404nm, with the Q wave between 500-700nm. The absorption peak of HCeC was similar to Ce6 besides a little red shift, which indicated that Ce6 had been successfully covalently linked to HCe. To explore the stability of nanomaterial, HCe and HCeC were mixed in a series of physiological solutions (water, PBS, DMEM, DMEM+10% FBS) for 24 h. As shown in **Supplementary Figure 2E**, we found there were no obvious agglomeration and precipitation of HCe and HCeC. These results indicated that HA modification could improve the stability of CeNPs effectively as literature report (51, 52). Besides, we found that the mass ratio of Ce to Ce6 of the HCeC is about 1:0.33 **Supplementary Figures 2F, G** and the HA modification could not affect the photosensitivity of HCeC in **Figure 1H**. The HCeC showed high CAT-like activity in **Figures 1I, J**. Therefore, we demonstrated that HA had been successfully modified on the surface of nano-sized cerium oxide and covalently bound to Ce6, which provided a foundation for further biological application.

### 3.2 Characterization of the Gel-HCeC-CaO_2_ system

**Figure 2:**
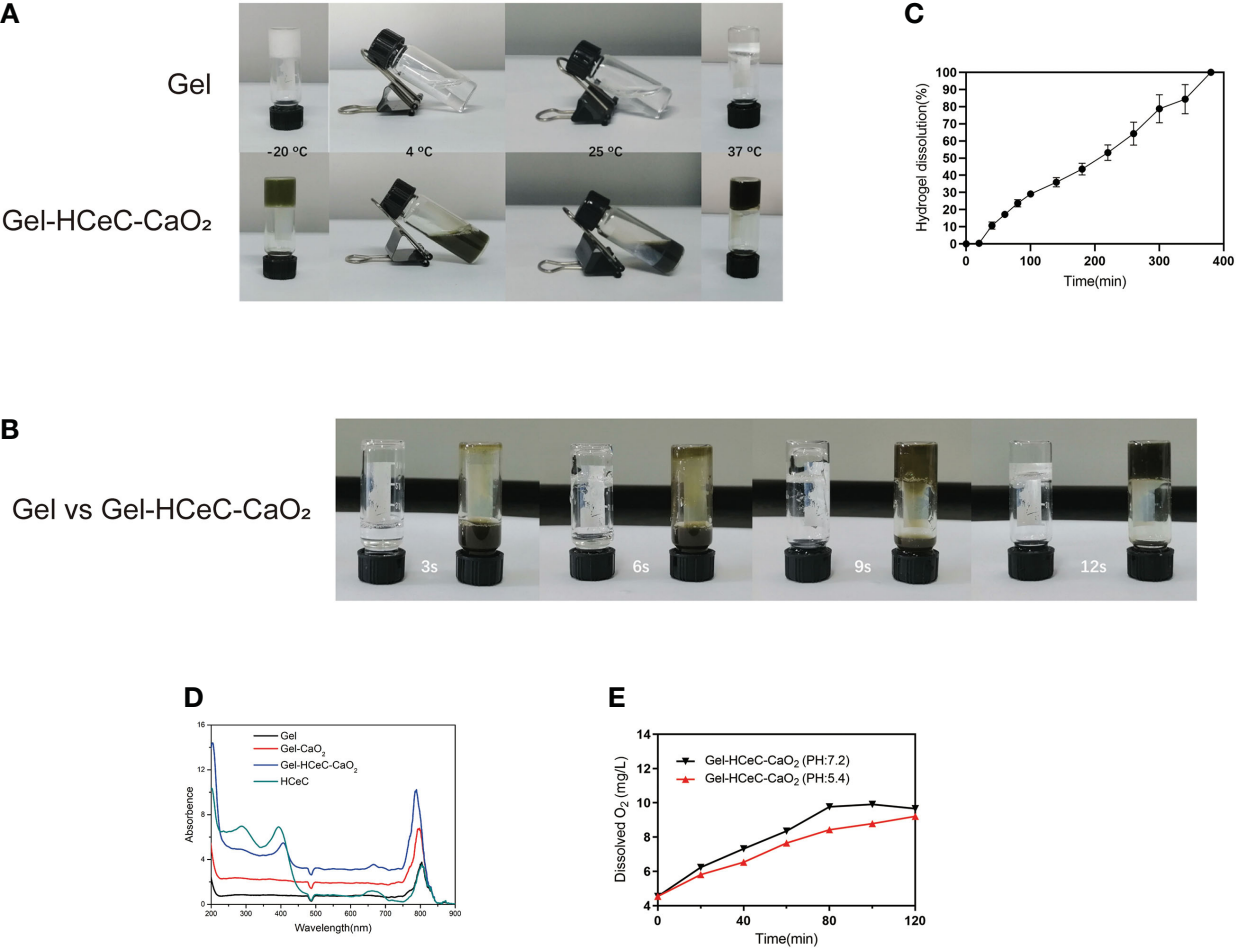
Characterization of the Gel-HCeC-CaO_2_ system. **(A)** The sol-gel transition behavior of Gel and Gel-HCeC-CaO_2_ system. **(B)** The gelation time of Gel and Gel-HCeC-CaO_2_. **(C)** The corrosion behavior of the Gel-HCeC-CaO_2_. **(D)** The photophysical properties of HCeC, Gel, Gel-CaO_2_, and Gel-HCeC-CaO_2_. **(E)** The O_2_ generation of Gel-HCeC-CaO_2_.

Hydrogel was a kind of polymer material that had been widely studied at present (53), which could be converted between liquid and solid state with a change of temperature. To show the sol-gel transition behavior of the hydrogel by the test tube inverting method (44), the Gel and Gel-HCeC-CaO_2_ were liquid at -20°Cˎ 4°C ˎ25°Cand 37°C. As shown in **Figure 2A**, the liquid transformed into gel at -20°C and 37°C and the sol state at 4°C and 25°C. With the extension of the bath time at 37°C, the hydrosol transformed into the gel phase gradually, and the rheological temperature was affected by the addition of CaO_2_ and HCeC. The gelation time of Gel-HCeC-CaO_2_ was approximately 9 s with the Gel needing 12 s and more time in **Figure 2B**. To observe the corrosion behavior of the Gel, the release experiment *in vitro* was performed, which showed the gel is almost completely dissolved at approximately 6 h in **Figure 2C**, following zero-order kinetics. Besides, the UV–vis absorption spectroscopy was used to investigate the photophysical properties of HCeC, Gel, Gel-CaO_2_, and Gel-HCeC-CaO_2_ in **Figure 2D**, and the absorption peak of Gel-HCeC-CaO_2_ was similar to HCeC, indicating that HCeC was mixed with the gel.

Furthermore, owing to the catalase activity of HCeC, the O_2_ generation of Gel-HCeC-CaO_2_ *in vitro* was detected. As shown in **Figure 2E**, the generation rate of oxygen from Gel-HCeC-CaO_2_(PH=7.2) was fast compared with other groups, indicating that the catalase activity of HCeC was influenced by the PH value. Owing to the catalase activity of the Gel-HCeC-CaO_2_ being affected by PH (54), the catalase activity of the Gel-HCeC-CaO_2_ was the highest under neutral conditions while reducing the catalase activity under acidic conditions. Therefore, the Gel-HCeC-CaO_2_ could release oxygen slowly to relieve tumor hypoxia in the acidic tumor microenvironment.

### 3.3 *In vitro* PDT of HCeC and Gel-HCeC-CaO_2_ system

**Figure 3:**
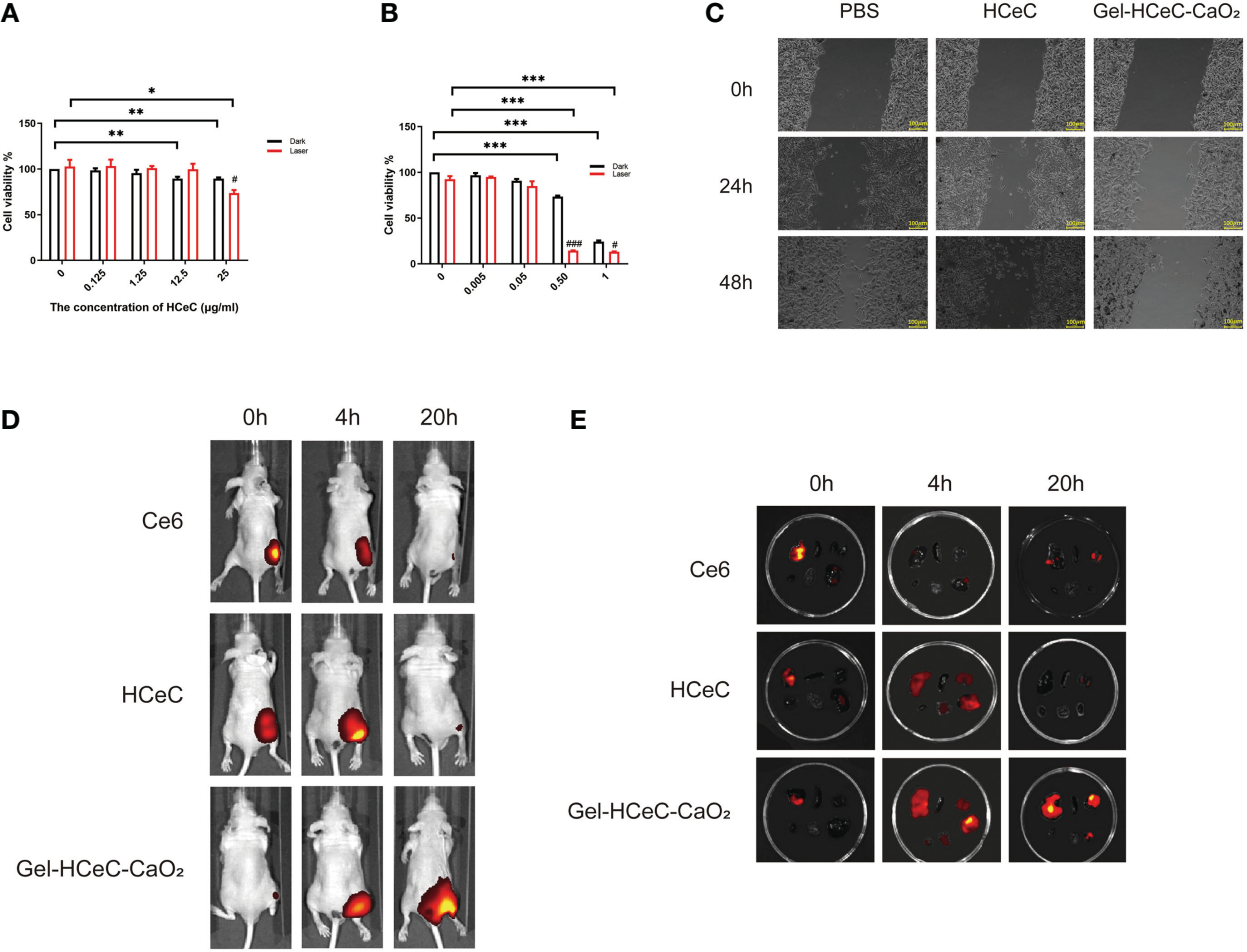
*In vitro* photothermal of HCeC and biodistribution of Gel-HCeC-CaO_2_ system. **(A)** The phototoxic effect of HCeC. **(B)** The phototoxic effect of Gel-HCeC-CaO_2_ system. **(C)** The migration of B16F10 cells treated with Gel-HCeC-CaO_2_ (Laser). **(D)** The biodistribution of Gel-HCeC-CaO_2_ *in vivo*. **(E)** Fluorescence imaging of some organs (Top: liver, spleen, kidney; Bottom: heart, lung, tumor). ∗p < 0.05; ∗∗p < 0.01; ∗∗∗p < 0.001.

To detect the phototoxicity of Gel-HCeC-CaO_2_, cell viability was measured by CCK-8. There was almost no significant phototoxic effect observed in B16F10 cells treated with HCeC with or without irradiation in **Figure 3A**. However, the B16F10 cells treated with Gel-HCeC-CaO_2_ (Laser) had a significant effect on B16F10 cells compared with Gel-HCeC-CaO_2_ (Dark) in **Figure 3B**. Because the Gel-HCeC-CaO_2_ (Laser) could release oxygen to improve the PDT efficiency, and produce a large number of ^1^O_2_ and ROS to kill tumor cells. Moreover, the H_2_O_2_ (300 μM) could almost kill 50% of B16F10 cells owing to the toxicity in **Supplementary Figure 3A**, but the H_2_O_2_ (100 μM) had almost no effect, confirming that the toxicity of Gel-HCeC-CaO_2_ was not caused by H_2_O_2_. To test the migration of B16F10 cells treated with Gel-HCeC-CaO_2_ (Laser), we performed the scratch test. As shown in **Figure 3C**, the migration ability of B16F10 cells in the Gel-HCeC-CaO_2_ (Laser) group was significantly lower compared with other groups. Therefore, the Gel-HCeC-CaO_2_ (Laser) could enhance PDT efficiency and kill B16F10 cells significantly.

### 3.4 *In vivo* biodistribution of Gel-HCeC-CaO_2_ system

To show the biodistribution of Gel-HCeC-CaO_2_ *in vivo*, the fluorescence of Ce6, HCeC, and Gel-HCeC-CaO_2_ in mice was detected at 0 h,4 h, and 20 h post-injection. As shown in **Figure 3D**, only a small amount of fluorescence appeared at the tumor site in Ce6 and HCeC group whereas predominantly observed in the Gel-HCeC-CaO_2_ group at 20 h, suggesting that the good retention of the Gel-HCeC-CaO_2_ in the mice. The biodistribution of the nanomaterials in mice was quantitatively analyzed in **Figure 3E**. Fluorescence imaging of some organs like lungs, liver, and kidneys showed that free Ce6 and HCeC were rapidly cleared over time up to 20 h, and the Gel-HCeC-CaO_2_ had the good retention of the tumor to improve the PDT efficiency.

### 3.5 *In vivo* photodynamic effect of Gel-HCeC-CaO_2_ system

**Figure 4:**
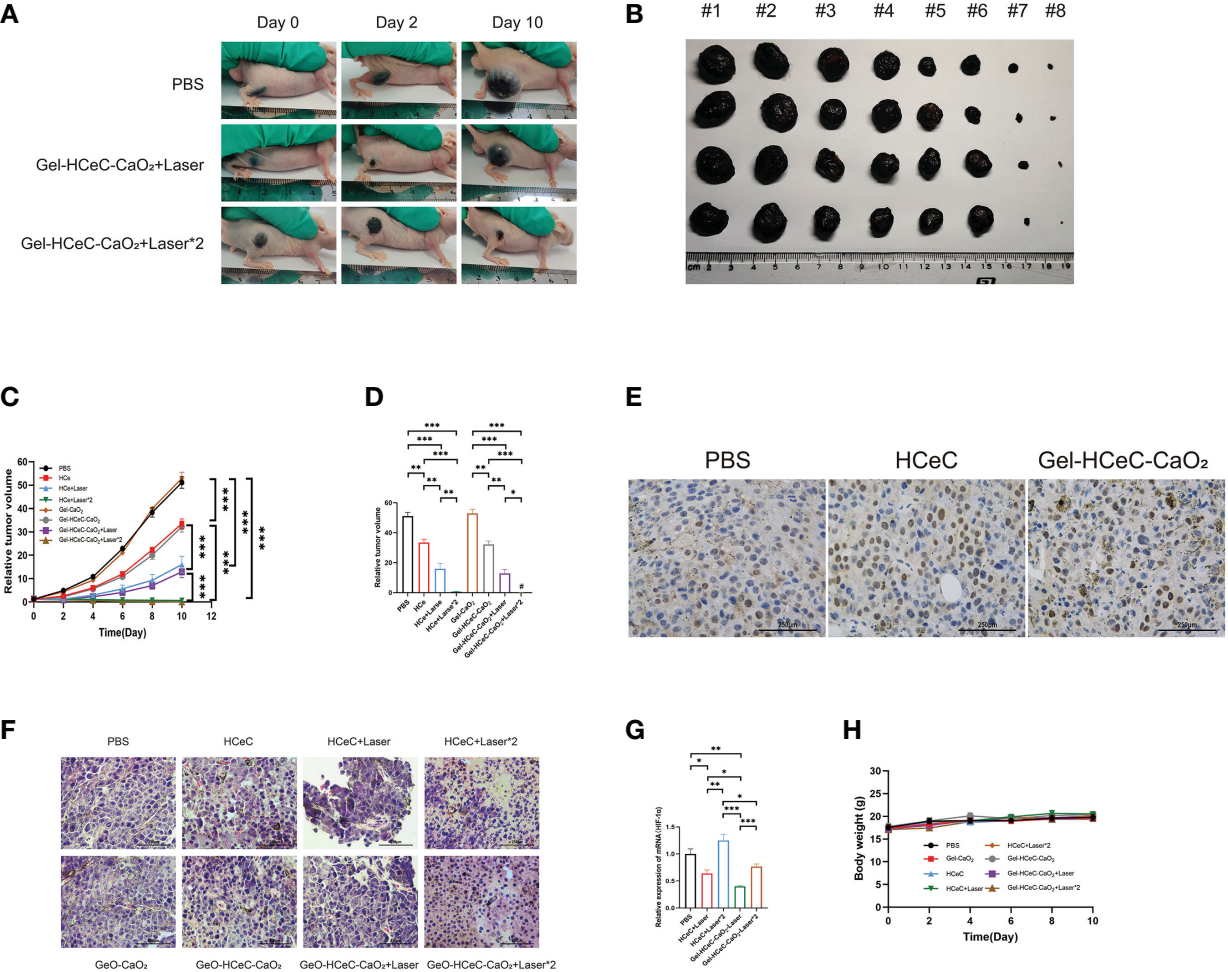
*In vivo* photothermal effect of Gel-HCeC-CaO_2_ system. **(A, B)** The tumors of mice in each group were photographed (#1: PBS; #2: Gel-CaO_2_; #3: HCeC; #4: Gel-HCeC-CaO_2_; #5: HCeC+Laser; #6: Gel-HCeC-CaO_2_+Laser; #7: HCeC+Laser*2; #8: Gel-HCeC-CaO_2_+Laser*2). **(C, D)** The HCeC and Gel-HCeC-CaO_2_ showed significant tumor regression with a reduction of the tumor volume. **(E)** Tunnel assay of the tumor. **(F)** HE staining of tumor. **(G)** The expression of HIF-1 α. **(H)** The body weight of mice. *p < 0.05; **p < 0.01; ***p < 0.001.

The athymic nude mouse xenograft B16F10 model was generated for photodynamic therapy of melanoma. To examine the PDT efficiency of different materials to reduce tumor progression in the athymic nude mouse xenograft B16F10 model, 100μL PBS, Gel-CaO_2_, HCeC, Gel-HCeC-CaO_2_ were intravenously injected into the mice at 1 week after engraftment, and the NIR irradiation (660 nm, 200 mW/cm^2^, 5 min) was administered to the tumor site at 4 h and 20 h post-injection in the laser groups in **Supplementary Figures 3B, C**. The tumors of mice in each group were photographed, the Gel-HCeC-CaO_2_ group could significantly inhibit the B16F10 tumor progression in **Figures 4A, B**. As shown in **Figures 4C, D**, the HCeC and Gel-HCeC-CaO_2_ groups showed significant tumor regression with a reduction of the tumor volume by ∼42%, indicating the chemotherapy toxicity of the HCeC. The laser groups which irradiated one time could delay B16F10 tumor progression by∼69%, indicating that HCeC had obvious phototherapy toxicity. The Gel-HCeC-CaO_2_(Laser*2)had high tumor growth inhibition than HCeC(Laser*2), showing that Gel-HCeC-CaO_2_ could satisfy repeat PDT therapy.

The tunnel assay confirmed that the Gel-HCeC-CaO_2_ (Laser) group induce apoptosis of B16F10 cells *in vivo* in **Figure 4E**. Furthermore, hematoxylin and eosin (H&E) staining revealed large areas of necrosis, inflammatory cell infiltration and broken blood vessels of tumor tissue in Gel-HCeC-CaO_2_ (Laser*2) group in **Figure 4F**. Hypoxia-inducible factor 1 α (HIF-1 α) was an important transcriptional regulator, which was crucial for tumor progression (55). The Gel-HCeC-CaO_2_ system could significantly inhibit the expression of HIF-1 α compared with the HCeC in **Figure 4G**, indicating that Gel-HCeC-CaO_2_ could alleviate the hypoxia of the tumor microenvironment. Besides, there were no metastatic tumor nodules observed in the lungs of the mice in **Supplementary Figure 3D**. To show the systemic toxicity of materials, we observed the body weight and histological change of mice. As shown in **Figure 4H**, the treatment could not change the body weight of mice. Therefore, the Gel-HCeC-CaO_2_ (Laser) could reduce tumor progression in the athymic nude mouse xenograft B16F10 model significantly.

## 4 Discussion

**Figure 5:**
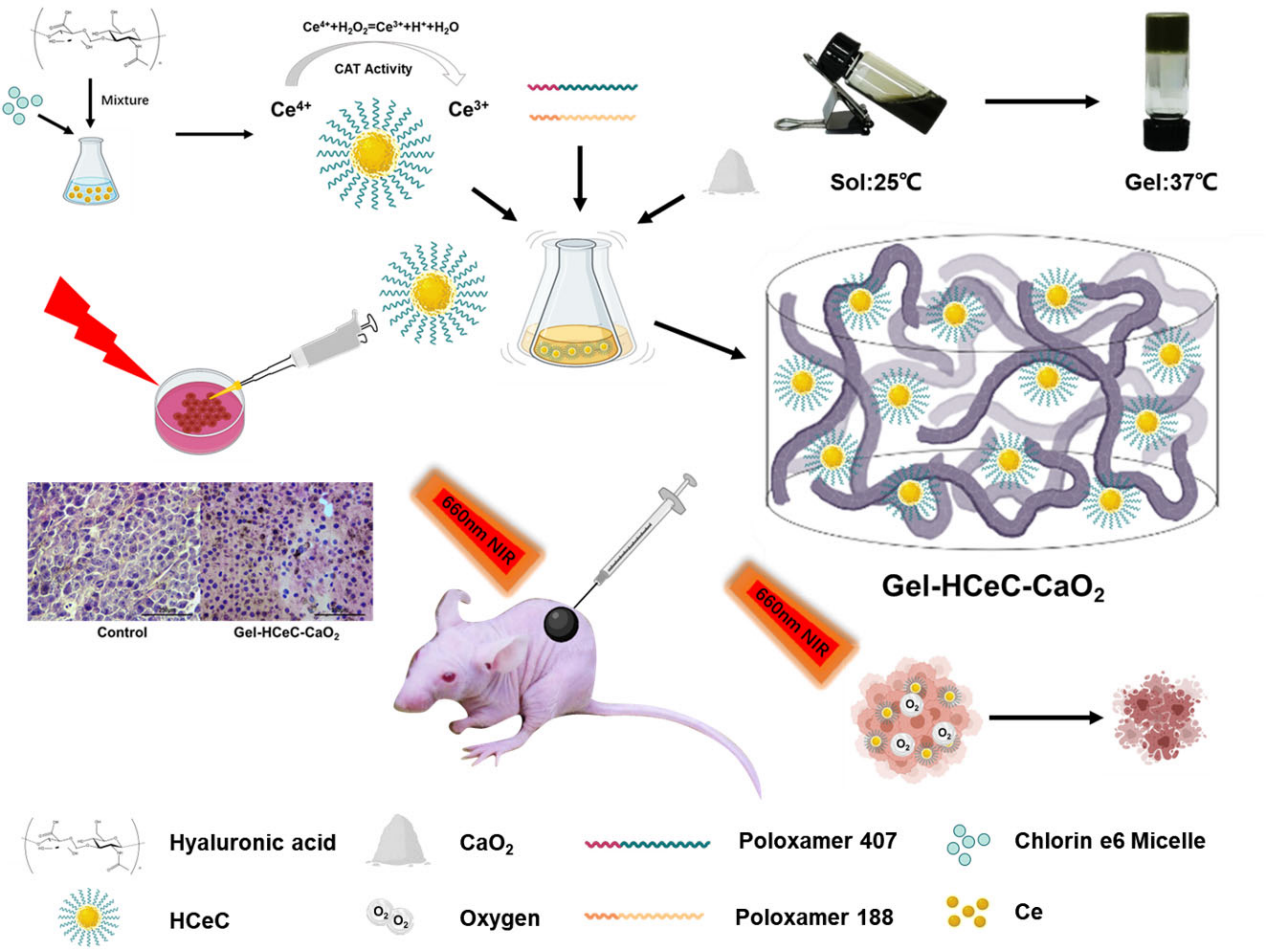
Synthesis of the Gel-HCeC-CaO_2_ system. The Gel-HCeC-CaO_2_ system could alleviate tumor hypoxia microenvironment, prolong drug supply, enhance PDT efficacy, and overcome melanoma.

Notably, the inadequate amount of oxygen generation and short-term effects of photosensitizers induced by drug metabolism and clearance in the tumor environment cannot ameliorate the aggravated stuff supply mediated by PDT consumption (10–12), failing efficient and repeated PDT. Therefore, an efficient long-term oxygen supply and drug delivery system should be explored for PDT efficacy improvement and tumor inhibition. Herein, a prolonged oxygen-generating phototherapy hydrogel system (Gel-HCeC-CaO_2_) was designed to alleviate tumor hypoxia, prolong drug supply, enhance PDT efficacy, and overcome melanoma. As shown in **Figure 5**, PSs loaded biocompatible CeNPs nanocarrier (HCeC) was synthesized by an HA-mediated self-assembly method under an alkaline environment, followed by incorporation into a thermo-sensitive hydrogel prepared by simple mixing Pluronic^®^ F127 and F68, the FDA-approved polymers (42). Sufficient oxygen generation was obtained by introducing an O_2_-evolving agent CaO_2_ into the hydrogel system, which could react with H_2_O to form H_2_O_2_ and followed produce O_2_ catalyzed by catalase-like CeNPs. The prolonged oxygen supply was achieved by the prepared hydrogel. The prepared hydrogel could limit the infiltration of H_2_O into the system and moderate the hydrolysis rate of CaO_2_, resulting in sustained hypoxia alleviation in tumor tissues. Besides, the prepared hydrogel could act as a sustained delivery system and be easily injected around the tumor, resulting in prolonged drug accumulation. The accumulated HCeC entered into tumor cells by endocytosis mediated by CD44, a targeted receptor of HA overexpressed on most of the malignant tumors, resulting in a promoted PSs cellular uptake. Synthetically, a “once injection, repeat irradiation” strategy was achieved through the developed hydrogel system to enhance the PDT efficiency and overcome melanoma.

## 5 Conclusion

In this study, we prepared a Gel-HCeC-CaO_2_ system which showed a significant phototoxic effect of B16F10 tumor by NIR irradiation. The Gel-HCeC-CaO_2_ system showed the following advantages: (i) utilized the catalase activity of HCeC to produce O_2_; (ii) good retention of the HCeC in the tumor; (iii) no severe systemic toxicity; (iv) alleviate tumor hypoxia environment, meeting the repeated photodynamic therapy strategy and effectively inhibiting tumor metastasis. Therefore, we believe the Gel-HCeC-CaO_2_ system will provide a new strategy for the treatment of melanoma, which greatly alleviates the tumor hypoxia microenvironment to improve PDT efficiency.

## Data availability statement

The original contributions presented in the study are included in the article/**Supplementary Material**. Further inquiries can be directed to the corresponding author.

## Ethics statement

The animal study was reviewed and approved by Centre for Diseases Prevention and Control of Eastern Theater.

## Author contributions

LZ and XL had contributed equally to this work. All authors approved the final manuscript. HL, LZ, and XL designed the study and instructed all experiments. LZ and XL carried out the data analysis and drafted the manuscript. SR, YC, and ZC assisted in performing the experiments. YM, YQ, and HL provided many suggestions on the articles and obtained funding. All authors contributed to the article and approved the submitted version.
