# Supplementary material for: Inhibition of melanoma using a nanoceria-based prolonged oxygen-generating phototherapy hydrogel
Source: Front Oncol. 2023 Mar 16;13:1126094. doi: 10.3389/fonc.2023.1126094 (PMC10060878; doi:10.3389/fonc.2023.1126094)
Supplement: Supplementary file 1 [file DataSheet_1.docx]

***Supplementary Material***

**1.1 Supplementary Figures**

**1.1.1 Supplementary Figure 1**

**
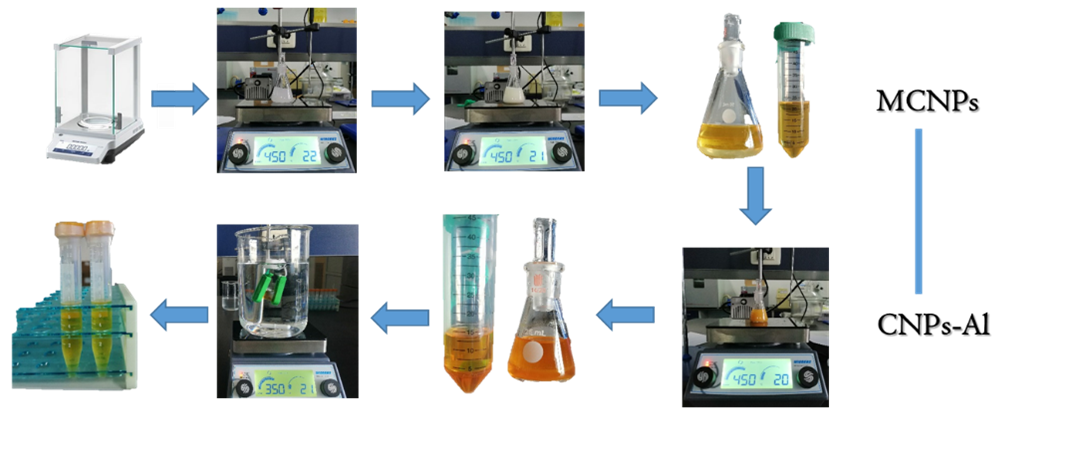
**

**Supplementary Figure 1.** The synthesis of MCNPs and CNPs-Al.

**1.1.2 Supplementary Figure 2**

**
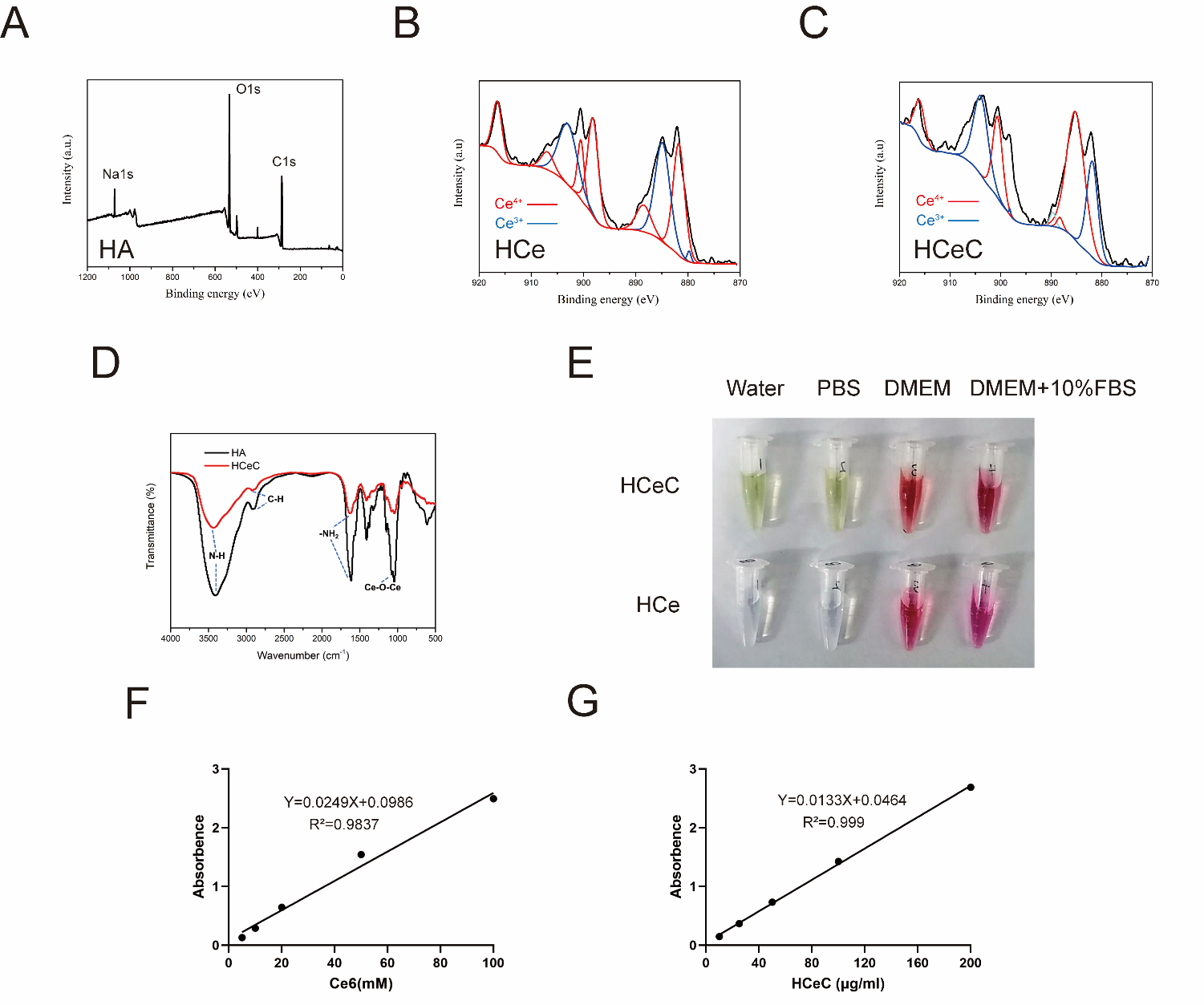
**

**Supplementary Figure 2.** Characterization of HCe and HCeC. (A) XPS spectra of HA. (B) Valence analysis of Ce 3d of HCe. (C) Valence analysis of Ce 3d of HCeC. (D) FT-IR spectra of HA and HCeC. (E) The stability of HCe and HCeC. (F-G) The mass ratio of Ce to Ce6 of the HCeC.

.

**1.1.3 Supplementary Figure 3**

**
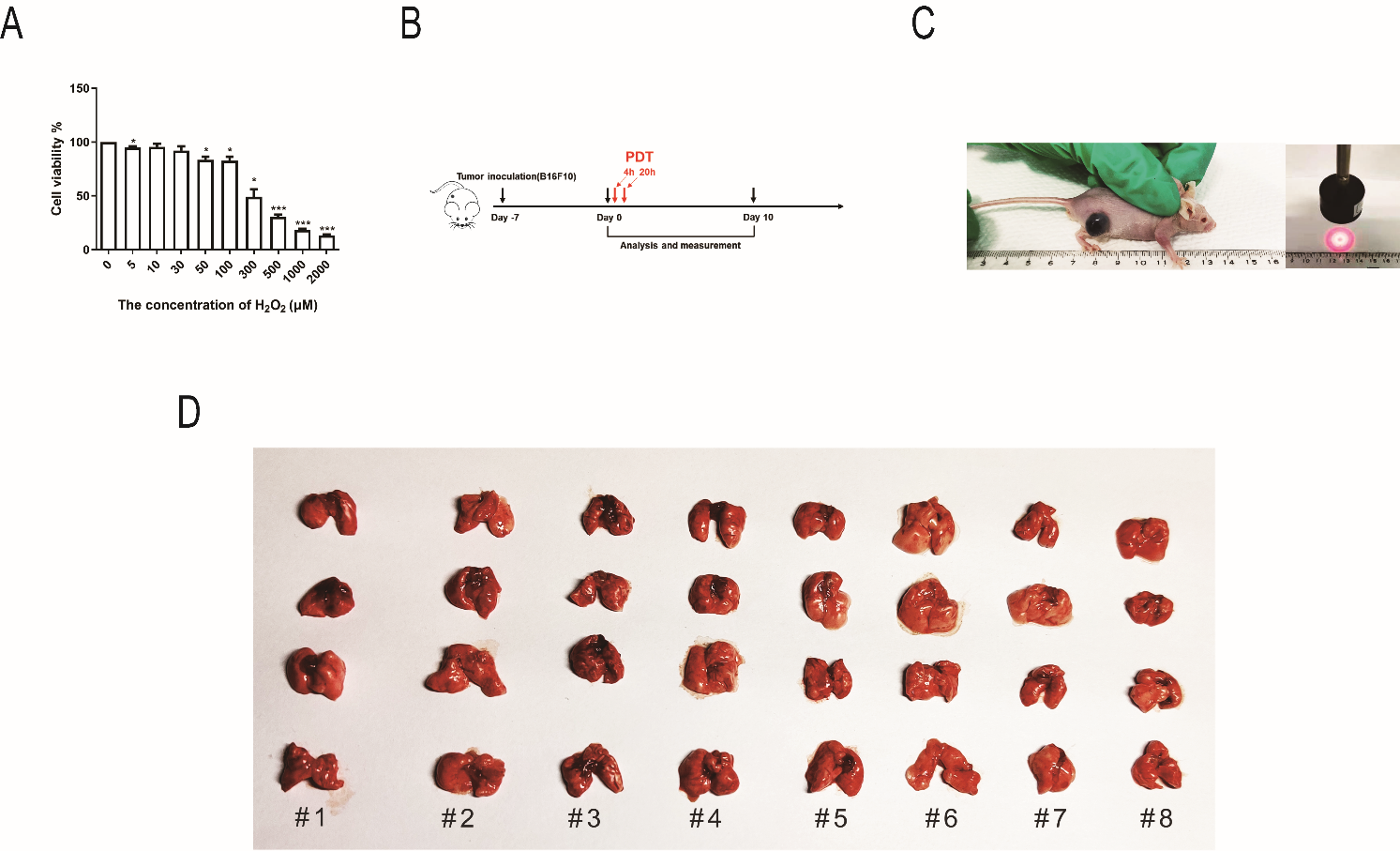
**

**Supplementary Figure 3.** The photothermal effect of Gel-HCeC-CaO_2_ system. (A) The toxicity of H_2_O_2_. (B-C) The athymic nude mouse xenograft B16F10 model was generated for photodynamic therapy of melanoma. (D) The tumor nodules were observed in the lungs (#1: PBS; #2: Gel-CaO_2_; #3: HCeC; #4: Gel-HCeC-CaO_2_; #5: HCeC+Laser; #6: Gel-HCeC-CaO_2_+Laser; #7: HCeC+Laser*2; #8: Gel-HCeC-CaO_2_+Laser*2)
